# Exceptional responses to Trastuzumab deruxtecan in HER2-positive breast cancer: two illustrative case reports

**DOI:** 10.3389/fonc.2025.1647018

**Published:** 2025-10-22

**Authors:** Zelmira Ballatore, Helene Vanacker, Rita Chiari, Thomas Bachelot

**Affiliations:** ^1^ UOC Oncologia, Azienda Sanitaria Territoriale Pesaro Urbino, Urbino, Italy; ^2^ Department de Cacerologie Medicale, Centre Leon Berard, Lyon, France

**Keywords:** breast cancer, HER2 breast cancer +, Trastuzumab deruxtecan (T-DXd), antibody drug conjugate (ADC), HER - 2/neu

## Abstract

**Background:**

Trastuzumab deruxtecan (T-DXd), an antibody-drug conjugate targeting human epidermal growth factor receptor 2 (HER2), has demonstrated substantial clinical activity in metastatic HER2-positive breast cancer. Although durable responses are increasingly reported, the potential for long-lasting remission after treatment discontinuation remains poorly documented.

**Cases:**

We report two patients with metastatic HER2-positive breast cancer who experienced exceptional and sustained clinical benefit with T-DXd. The first patient achieved a deep response lasting over 50 months, including 36 months of complete remission after treatment discontinuation due to thrombocytopenia. The second patient has maintained disease control for over 39 months, with a prolonged treatment pause. Both cases highlight remarkable disease stability beyond conventional expectations.

**Conclusion:**

These case reports highlight the potential of T-DXd not only to induce deep and durable responses but also to maintain disease remission beyond drug exposure in selected patients. Such observations challenge the paradigm of incurability in metastatic HER2-positive breast cancer and warrant further investigation into the mechanisms and predictors of long-lasting responses.

## Background

Breast cancer (BC) is the first cause of malignancy and the leading cause of cancer-related death in women worldwide (1, 2).

Approximately 30% of all breast cancers overexpress or present an amplification of the human epidermal growth factor receptor 2 (HER2), a feature of biological aggressivity, high risk of relapse, and poor prognosis. The availability of the more recent anti-HER2-targeted therapies has significantly changed the course of Her2-positive diseases, improving relapse rate and increasing survival.

The first in class of anti-Her2 drug was trastuzumab approved in 1998, and in the last 20 years, more than eight products have been approved by the FDA.

Trastuzumab deruxtecan (T-DXd) is a new antibody drug conjugate (ADC) using a DNA topoisomerase I inhibition assay, with high chemo power which makes it an ideal payload candidate for ADC. Another important consideration is about the stability of the linker in plasma, which minimizes systemic toxicity. The drug-to-antibody ratio of T-DXd is approximately 8, the highest of any currently approved ADC (3).

T-DXd acts with a bystander antitumor effect, which occurs when the cytotoxic payload is released in the tumor cells, diffuses across membranes (due to the high membrane permeability of the payload), and then enters and kills neighboring tumor cells (4).

Trastuzumab deruxtecan due to the very innovative mechanism of action constitutes remarkable addition to the oncologist’s clinical arsenal for transforming the management of HER2-positive breast cancer (5, 6).

However, there are still many challenges in Her2-positive BC, including resistance to HER2-targeted therapies, the development of brain metastases, the need to determine the optimal sequence of anti-Her2, and the need to identify biomarkers to improve patient outcomes and personalized care (7).

The safety and tolerability profile of T-DXd are manageable with hematological and gastrointestinal adverse events as most frequently reported. There are no biomarkers to identify who will benefit most and who will develop toxicities such as interstitial lung disease (ILD). That is a rare but feared adverse event due to its possible evolution in severity and for which careful medical attention and patient education are cornerstone (8, 9).

Furthermore, there are no consistent literature data regarding the end of systemic therapy for those patients that achieved a complete response of disease, neither about the outcome and the safety of a rechallenge of T-DXd after suspension due to severe adverse events (10).

## Case series

Here, we described two very singular clinical cases about the therapeutic strategy of Her2-positive BC. All adverse events are graduated conforming to Common Terminology Criteria of Adverse Event (CTCAE) v5.

According to legislation, the medical record was reviewed to find data on patients’ medical history and a written informed consent was obtained from the patients for publication of medical histories and any accompanying images. A copy of the written consent is available for review by the Editor in Chief of this journal.

Our first case report is about a French woman born in October 1975; there are no relevant comorbidities in her medical history (**Table 1**).

**Table 1:** Sociodemographic and clinical features .

Variable	Clinical case 1	Clinical case 2
Age at primary breast cancer (y.o.)	39	42
Year of BC diagnosis	2015	2009
Age at metastatic breast cancer (y.o.)	40	49
Year of MBC diagnosis	2016	2016
Race/ethnicity	Caucasian	Caucasian
Basal ECOG PS	0	1
Location	France	Italy
Disease stage at diagnosis, no. (%)	IIIC	IIB
Disease-free interval	<6 months	>5 years
Before T-DXd treatment		
Prior pertuzumab	Yes	Yes
Prior TDM1	Yes	Yes
Prior lines of therapy	6	4

In June 2015 (39 years old), she was diagnosed with an inflammatory tumor of the right breast, non-specific type, with involvement of the left supraclavicular lymph nodes, cT4d N3c, according to the American Joint Committee on Cancer (AJCC, 8th Edition). The biological features at immunohistochemistry analysis showed negative hormonal receptors, high proliferative index (Ki67 25%), and Her2 score 3+. In July 2015, a baseline total body computed tomography (CT) scan was performed, and no distant metastases were found, and then she was treated with pertuzumab, trastuzumab, and docetaxel, for 6 cycles, with no relevant toxicities reaching local progression of disease as best response. Then, in October 2015 she was switched to trastuzumab emtansine that was received for 4 months, resulting in clinical and radiological partial responses of disease.

The benefit from TDM1 created the chance of a right mastectomy plus axillary dissection performed in April 2016. Definitive histology showed 2 cm of residual invasive carcinoma with a micropapillary component, hormonal receptors negative, HER2 positive score 3+ on immunohistochemistry with the presence of vascular emboli, and negative resection margins. Two out of five nodes were involved, posttreatment stage ypT2N1a, R0. Postoperative radiation was performed.

In September 2016, there was a clinical and radiological evidence of metastatic recurrence in the left axillary lymph nodes and onset of bone metastasis, the patient was rechallenged with TDM1 in the first-line setting and local palliative radiation of bone lesion (L2 and left axillary node, 50 Gy in 25 fractions).

There was a benefit lasting until January 2017 when she reported a local relapse in the right mastectomy area with the appearance of permeation nodules. At the total body CT scan, there was no evidence of other metastasis.

Due to the above-described progression of disease, a third-line chemotherapy with trastuzumab and capecitabine was administered from February until October 2017; the best response was stability of the disease. In October 2017, a worsening of disease showed an increasing number and extension metastasis in the chest skin and dorsal bone spine (D8-D9).

A fourth-line combination with trastuzumab plus vinorelbine was prescribed until June 2018, when she presented to the oncologic department with severe headache and nausea, highly suspected of intracranial hypertension. A brain MRI was performed showing a right parieto-occipital brain metastasis of 6 cm and moderate edema.

There were no other central nervous localizations of disease, and the case was discussed in the multidisciplinary team: It was considered surgery after steroid therapy. The tumorectomy analysis confirmed a negative status of hormonal receptors and the overexpression of HER2. A postsurgery stereotactic radiotherapy was performed for 30 Gy in six fractions, and she continued to receive maintenance with 3-weekly trastuzumab.

After approximately 6 months, in January 2019 due to a significant skin progression, the patient was included in the MERUS trial, a phase II, open-label, multicenter international study to evaluate the efficacy of MCLA-128-based combinations, and she was randomized to receive MCLA 128 (zenocutuzumab) plus trastuzumab. There was neither clinical nor instrumental benefit since she developed clinical cutaneous, node and lung progression of the disease at the first-schedule CT scan after two cycles of treatment.

In March 2019, she was included in a SOPHIA trial substudy (7) and she received margetuximab plus gemcitabine until October 2019, with a mixed response of cutaneous progression and stationarity of node, bone, and lung lesions. There was no active brain disease.

**Figure 1:**
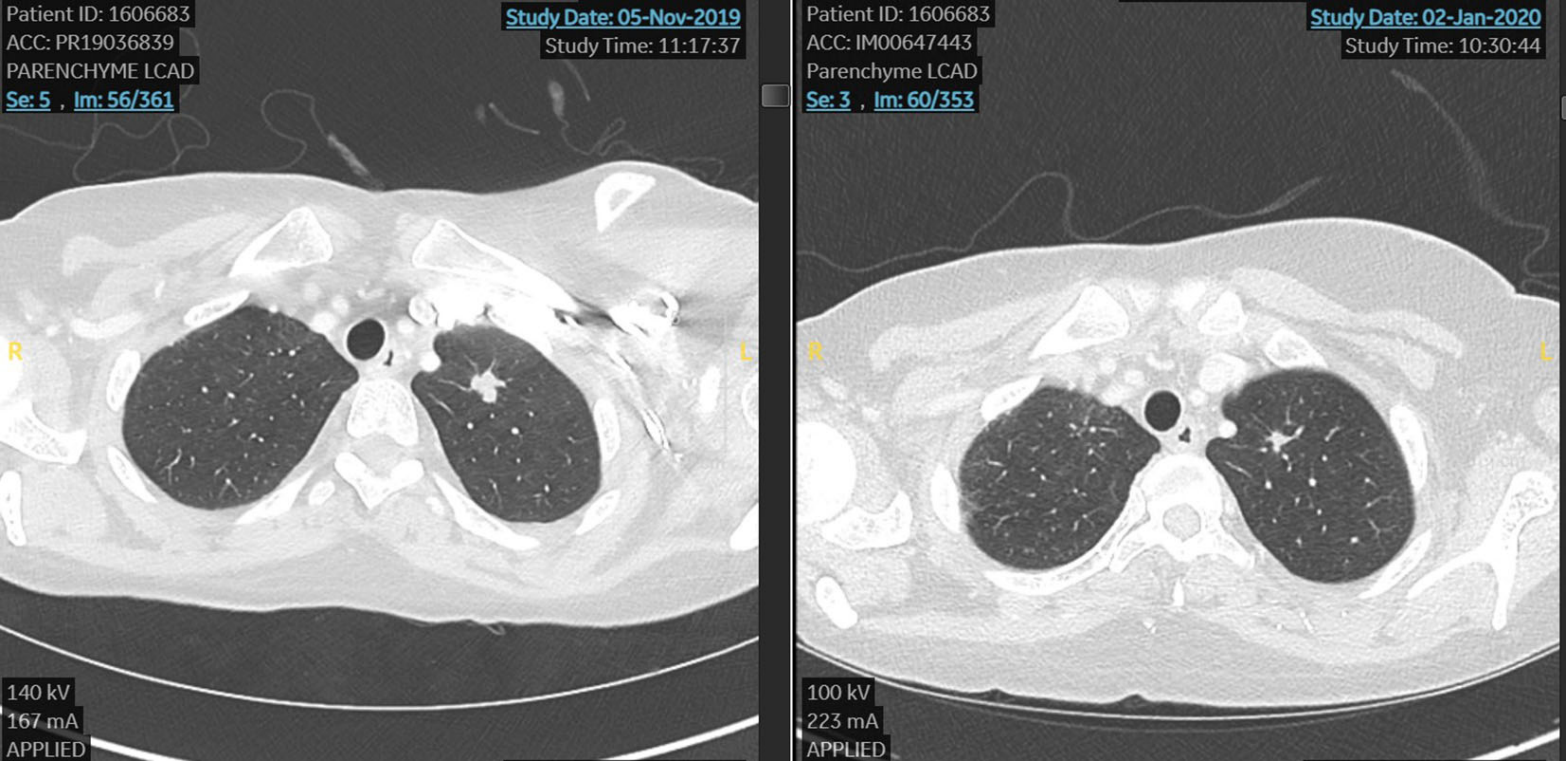
First case. Lung metastasis at the start and after 6 weeks of treatment with TDX-d (partial response by RECIST 1.1).

In November 2019 due to the bone, node, and lung progression disease, she was considered for a DAISY study and she was randomized to the experimental arm and treated with DS82 (trastuzumab deruxtecan). She reached a very early benefit in skin lesions (after 3 weeks) and a partial response by RECIST 1.1 at the first scan after 6 weeks of treatment (**Figure 1**).

In June 2020, the treatment was stopped due to poor tolerance. She reported grade 3 nausea despite aprepitant plus ondansetron and low doses of chlorpromazine; asthenia grade 3 and thrombocytopenia grade 2 for which multiple dose reductions were performed without benefit (T-DXd 4.4 mg/kg at cycle 6, and then 3.2 mg/kg from cycles 7 to 10). After the discontinuation of T-DXd, there was an onset of persistent grade 3 thrombocytopenia which lasted more than 2 months.

A chronic thrombocytopenia workup was conducted resulting in a normal bone marrow, and there was a clinical suspect of hypersplenism. Then, the patient underwent splenic embolization in May 2021 reaching a subsequent normalization of the platelet count. However, the patient refused to restart T-DXd and clinical and instrumental follow-up was pursued every 4 months from June 2020 up today, resulting in no evidence of recurrence of disease.

The last control was performed on January 20, 2025. The clinical medical history is summarized in **Table 2**.

**Table 2:** Clinical case number 1. .

Treatment line	Start and stop, year/month	Sites of metastasis at the start of treatment	Therapeutic schedule	Best response	TTP
1st	2015/7 to 2015/9	Locally advanced	Pertuzumab 420 mg day 1 plus trastuzumab 6 mg/kg day 1 plus docetaxel 80 mg/mq day1 w3 *	PD	3 months
2nd	2015/10 to 2016/2	Locally advanced	Trastuzumab emtansine 3.6 mg/kg day 1 w3	PR	4 months
	2016/9 to 2017/1	Nodes, bone	Trastuzumab emtansine 3.6 mg/kg day 1 w3	PD	4 months
3rd	2017/2 to 2017/10	Subcutaneous/skin, nodes, bone	Trastuzumab 6 mg/mq day 1 plus Capecitabine 2,000 mg/m^2^ orally twice a days 1–14 w3	SD	12 months
4th	2017/11 to 2018/6	Subcutaneous/skin, nodes, bone	Trastuzumab 6 mg/mq day 1 plus vinorelbine 30 mg/mq day 1, 8 w3	PD	8 months
	2018/6 to 2019/1	Subcutaneous/skin, nodes, bone	Trastuzumab 6 mg/mq day 1 w3	PD	6 months
5th	2019/1 to 2019/2	Subcutaneous/skin, nodes, bone, brain	Zenocutuzumab 750 mg plus trastuzumab 6 mg/kg w3	PD	2 months
6th	2019/3 to 2019/10	Subcutaneous/skin, nodes, bone, brain, lung	Margetuximab 15 mg/kg day 1 plus gemcitabine 1,000 mg/mq day 1, 8 w3	SD	6 months
7th	2019/11 to 2020/6	Subcutaneous/skin, nodes, bone, brain, lung	Trastuzumab deruxtecan 5.4 mg/kg w3	CR	Not reached

The second case is about an Italian woman born in October 1967, with an unremarkable medical anamnesis. She was a former smoker and declared a cancer family history (mother and grandmother were diagnosed with BC too). Genetic counseling and tests were done, but no germline mutations were detected (**Table 1**).

At the age of 42, at self-breast examination, she palpated a lump in her left breast and then an ultrasound confirmed the nodular lesion, measuring 2 cm in maximum diameter.

In December 2009, she underwent left breast-conserving surgery and ipsilateral axillary node dissection. The pathological report revealed grade 3 infiltrating ductal carcinoma, estrogen receptor (ER) positive (33%), progesterone receptor (PR) positive (20%), high proliferative index (Ki67 >30%), and Her2 score 3+. Three out of 19 axillary lymph nodes resulted positive. A total body CT scan was performed, and no distant metastases were found. The conclusion was stage IIB BC, pT2 (2.1 cm) pN1a (3/19), according to the American Joint Committee on Cancer (AJCC, 8th Edition).

In January 2010, adjuvant systemic chemotherapy was started and the patient received 3 cycles of the combination of 5-fluorouracil, epirubicin, and cyclophosphamide (FEC), followed by 3 cycles of docetaxel plus trastuzumab. Then, she completed standard radiation therapy and 1 year of trastuzumab.

After chemotherapy, due to the premenopausal status and hormonal receptor positivity, she received tamoxifen and gonadotropin-releasing hormone agonist for 5 years, until 2015.

There was no evidence of relapse of disease until June 2016, when the patient started complaining of mild dizziness and moderate headache. A brain, chest, and abdomen CT scan was performed showing multiple brain and lung metastases. She was treated with gamma-knife cerebral radiosurgery for four cerebellar lesions, reporting mild walking instability as an adverse event.

From July 2016 to August 2017, first-line chemotherapy was performed with docetaxel combined with pertuzumab and trastuzumab (7 cycles), followed by maintenance. The patient refused endocrine therapy in association to anti-HER2 therapy.

In October 2017, a local recurrence was diagnosed with no evidence of distant metastasis, then the breast multidisciplinary team decided for breast-conserving surgery. The histological report showed an infiltrating ductal carcinoma, grade 3, ER positive (10%), PR positive (5%), high proliferative index (Ki67 = 35%), and Her2 score 2+with positive *in situ* hybridization.

One month later, in November 2017, according to guidelines she started the second-line systemic therapy with trastuzumab emtansine until February 2019, when a whole-body CT scan highlighted a dimensional increase of lung lesions, the onset of multiple mediastinal nodes, and a new singular brain metastasis.

The large new cerebellar vermis lesion was treated with neurosurgery, and the histological report confirmed breast cancer origin, ER positive (10%), PR negative (0,1%), high proliferative index (Ki-67 = 52%), Her2 score 2+with positive *in situ* hybridization.

In March 2019, whole-brain radiation therapy was done (3,000 cGy over 12 fractions).

In April 2019, the patient was eligible for the SOPHIA clinical trial, a randomized, open-label, phase III trial evaluating margetuximab+chemotherapy vs. trastuzumab+chemotherapy of physician choice in patients with pretreated HER2+ metastatic BC; prior brain metastases were allowed if treated or stable.

She was randomized to the experimental arm and received margetuximab 15 mg/kg Q3W plus vinorelbine. The best response was a partial one by RECIST 1.1, reached after 3 cycles of treatment and maintained until July 2020. During treatment, she experienced a mild infusion reaction related to margetuximab at the first administration, resolved with temporary suspension of infusion and a recurrent mild transaminase increase (G1 vinorelbine related) during subsequent cycles.

In August 2020, the scheduled CT scan showed progression of disease by RECIST 1.1 with skin local recurrence, onset of peritoneal carcinosis, stability of lung metastasis, and a new central nervous system secondary lesions; she subsequently received gamma-knife for left fronto-opercular and cerebellar metastases.

From September 2020 to March 2021, capecitabine plus lapatinib was administered with stable disease as the best response and mild hand–foot syndrome (Grade 1 capecitabine related) as the adverse event. In the last 2 months of fourth-line treatment, there was a mild and progressive deterioration of clinical condition (ECOG 2) and the onset of gastrointestinal symptoms with severe constipation and left breast/axillary pain, whereby the patient received best supportive care.

**Figure 2:**
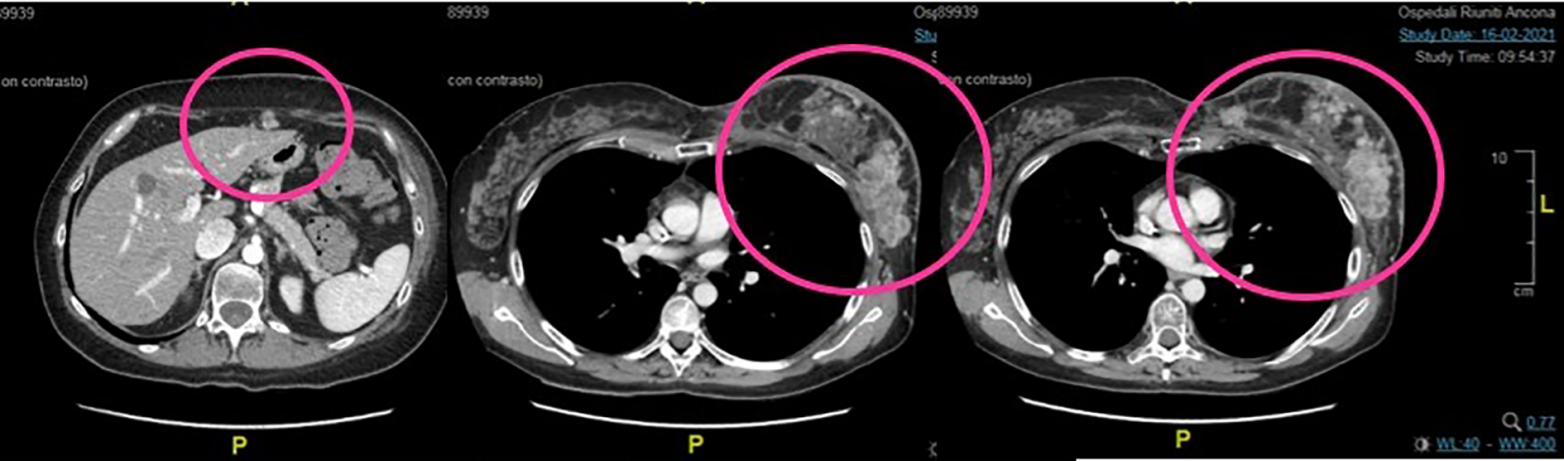
Second case. Baseline total body CT scan at the start of the treatment with TDX-d. There are evident peritoneal carcinosis, multiple subcutaneous lesions in the chest wall, and breast recurrence.

In February 2021, a total body CT scan confirmed the high clinical suspicion of progressive disease with peritoneal carcinosis, multiple subcutaneous lesions in the chest wall (**Figure 2** **),** multiple nodes, and lung metastasis. Taking into account the absence of available standard therapies and of further recruiting clinical trials and the patient request, we started the procedure to the expanded access program of trastuzumab deruxtecan (T-DXd).

**Figure 3:**
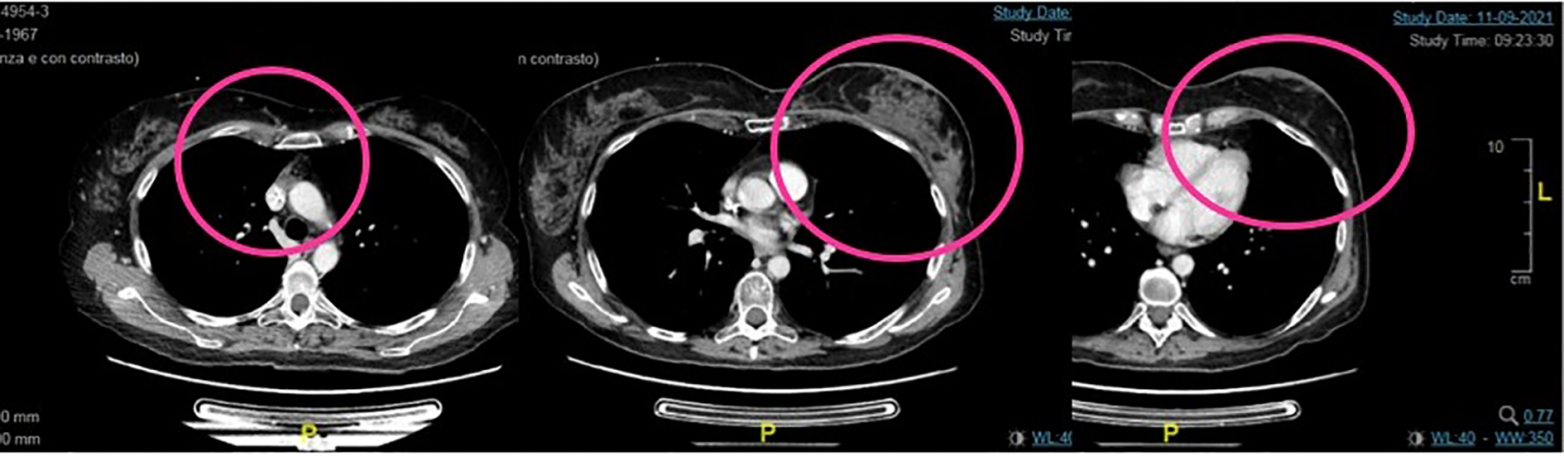
Second case. Complete response by RECIST 1.1 after 6 months of treatment with TDX-d.

The fifth line with T-DXd treatment was administered from April 2021 to January 2023, with partial response at the first instrumental evaluation in June 2021 and complete response at the second one in September 2021 (**Figure 3**).

**Figure 4:**
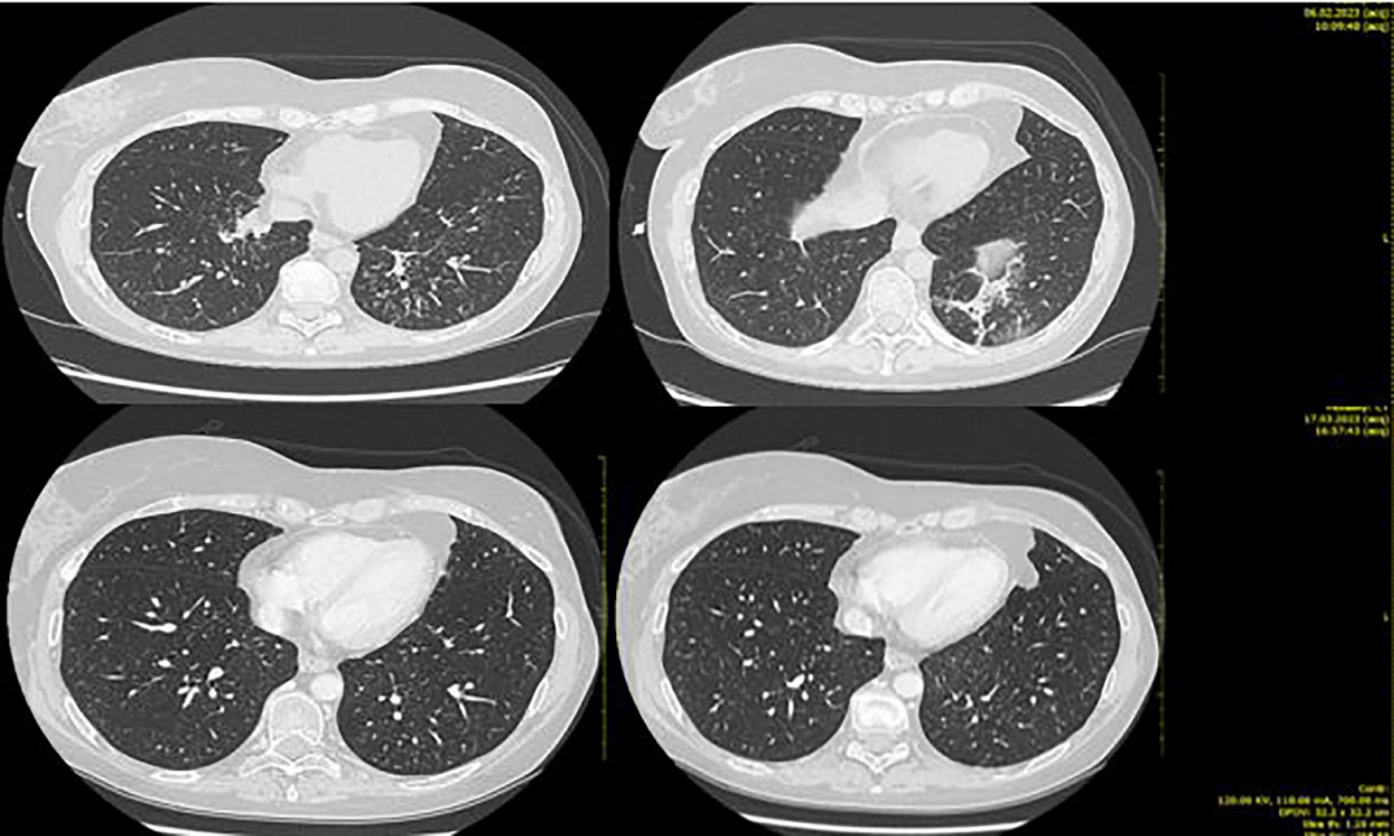
Second case. Bilateral ILD with greater involvement of basal left lung (upper part of the figure) and ILD initial resolution after high-dose steroid therapy (lower part).

Clinical and radiological benefits lasted until 6 of February 2023 when the patient came to the scheduled visit in ECOG PS 2, reporting new-onset moderate fatigue, moderate cough, and mild dyspnea. Vital signs were in range in the exception of oxygen saturation level that was borderline (94% in ambient area). An emergency chest CT scan showed thickening of the centrilobular and interlobular pulmonary interstitium bilaterally, with associated “tree-in-bud” aspects testifying for bilateral interstitial lung disease grade 3 trastuzumab deruxtecan related (**Figure 4**).

The patient was hospitalized and treated with high flow oxygen therapy, along with infusion of methylprednisolone 1,000 mg/day for 5 days with the addition of broad-spectrum prophylactic antibiotic therapy (levofloxacin 500 mg/day for 10 days).

During hospitalization, there was a progressive improvement in the general clinical conditions with resolution of cough and dyspnea and progressive improvement in thoracic clinic objectivity from the third day of steroid therapy. From 11 of February, the dosage of the infusional steroid was reduced to methylprednisone 1 mg/kg/day (as per ILD trastuzumab deruxtecan guidelines). On February 13, a chest CT showed a marked reduction in phlogosis (**Figure 4**).

T-DXd was discontinued due to the severity of the adverse event, and on 8 of March 2023, a total body CT scan showed complete resolution of the pulmonary thickening and no evidence of metastases. Taking into account the multiple treatments and the severity of the reported toxicities, clinical laboratory and instrumental follow-up was proposed.

From June 2023 up today, semestral brain MRI has been performed confirming the absence of central nervous system relapse.

In July 2023, a total body CT scan showed a hypervascular pseudonodular image of the skin of the left chest wall.

Due to the new suspected element, ultrasound and needle aspiration were performed and cytological and histological examination confirmed breast cancer, hormonal receptor negative, ki67 40%, Her2 score 3+. Then, a new total body CT scan (September 2023) confirmed the unique site of recurrence at the left chest wall. The patient expressed a very strong request for mastectomy and the desire to not perform reconstruction. After exhaustive information about the high risk of the invasive procedure and a detailed preoperative evaluation for anesthesia, in November 2023, radicalization of the local left breast was performed. The histological report confirmed invasive grade 3 ductal carcinoma (maximum diameter 1.2 cm), hormone receptors negative, high proliferative index, and Her2 overexpression.

After surgery, follow-up has been done every 4-6 months, and up to today, the patient has no clinical or imaging signs of local and distant recurrence of disease (last CT scan January 2025). The clinical case is summarized in **Table 3**.

**Table 3:** Clinical case number 2.

Treatment line	Start and stop, year/month	Sites of metastasis at the start of treatment	Therapeutic schedule	Best response	TTP
1st	2016/7 to 2017/9	Brain, lung	Pertuzumab 420 mg day 1 plus Trastuzumab 6 mg/Kg day 1 plus Docetaxel 80mg/mq day1 w3 *, **	PR	14 months
2nd	2017/11 to 2019/2	Brain, mediastinal nodes	Trastuzumab Emtansine 3,6 mg/kg day 1 w3	SD	16 months
3rd	2019/4 to 2020/7	Brain, mediastinal nodes	Margetuximab 15mg/kg day 1 plus Vinorelbine 30mg/mq day 1, 8 w3	PR	12 months
4th	2020/9 to 2021/3	Brain, mediastinal nodes, lung, skin, peritoneum	Lapatinib 1250 mg days 1–21 plus Capecitabine 2000mg/m2 orally twice a days 1–14 w3	SD	4 months
5th	2021/4 to 2023/1	Brain, mediastinal nodes, lung, skin, peritoneum, subcutaneous	Trastuzumab Deruxtecan 5,4 mg/kg w3	CR	not reached

* Loading dose pertuzumab 840 mg and trastuzumab 8mg/kg 1st cycle.

** Seven combination cycles, then pertuzumab plus trastuzumab in maintenance.

## Conclusion

HER2-positive BC constitutes a more aggressive subtype with a faster rate of metastases than other breast tumors (11–13). Antibody–drug conjugates (ADC) are linked to alternating carriers and cytotoxic drugs resulting in maximizing therapeutic efficacy to the target.

T-DXd showed effectiveness against not only in Her2-positive breast and gastric cancer (14) but also in low Her2-positive breast cancer and is largely used in many countries for these tumors (15, 16).

To the best of our knowledge, these are the first cases of patients with Her2-positive BC who achieved a long disease control with T-DXd treatment and maintain the benefit after 2 years from discontinuation of the drug due to toxicities.

At the last exploratory analysis of Destiny Breast 03 in the T-DXd arm, 12.6% of patients experienced a complete response and 66.3% experienced a partial response; the median duration of response was 30.5 months in the exp arm (95% CI, 23.0 month to not estimable). Interestingly, the median OS was 52.6 months and almost half of the patients (45.7%) in the T-DXd group were progression free at 3 years and more than 40% were free at 4 years (13), but there are no data about maintenance of response after suspension due to adverse event and without active systemic treatment.

Interestingly, the common adverse event thrombocytopenia reported by the two patients during ADC therapy has previously been described as associated with improved survival outcomes in those patients compared with those without this specific toxicity (17).

In key breast cancer trials, grade ≥ 3 thrombocytopenia was observed in 5.7%-24.9% of individuals treated with T-DM1 and 4.3%-7.0% in those treated with T-DXd (15, 18–21).

The mechanism of thrombocytopenia induced by ADC seems to be secondary to selective toxicity to megakaryocytes leading to impairment of megakaryocyte differentiation and proplatelet formation, but there is no clear evidence of toxicity to hematopoietic stem cells, which is the proposed mechanism of conventional chemotherapy-induced thrombocytopenia (22, 23).

Since the production of endogenous thrombopoietin is constant regardless of platelet count, stimulating megakaryocyte growth, differentiation, and platelet production may be a potential solution to overcome the severe thrombocytopenia induced by ADC, rather than interruption or suspension of treatment (22, 23). Further research is needed on this topic.

Drug-related ILD was reported in 10%-15% of patients receiving T-DXd in destiny studies, and it was the most common cause of treatment discontinuation in the experimental arm. However, there was a very small percentage of grade 3 in ILD-reported cases with a median time of occurrence of the event that was 5.5 months, anyway mainly during the first year of the treatment. There were no known predictive biomarkers to preliminarily identified high ILD risk patients, but major risk factors associated with ILD development has been established: age older than 65 years, time since diagnosis longer than 4 years, lung comorbidities, and basal low oxygen saturation (less than 95%) (9, 24).

Our second patient presented ILD over the 12 months of T-DXd therapy, and considering the above described risk factor, the only one was the very long time from initial breast cancer diagnosis. The major limitation in the second case clinical management was that neither bronchoscopy nor bronchial lavage was performed, but at that time, there were no specific guidelines for ILD management. Current guidelines stress the importance of proactive management with patient education to signs and symptoms of ILD and to report any changes to the healthcare team immediately, but they are also recommended pulmonologist consultation, blood culture, bronchoscopy, and bronchial lavage, and finally prophylaxis against Pneumocystis jirovecii pneumonia (9, 24).

Our case reports are a clear example of those patients with high efficacy of T-DXd and interruption of treatment with no impact on oncologic outcomes. There is the need for more real-world data to learn about the underlying biological mechanisms of lasting complete response after discontinuation, but of the development of toxicities too and the chance of a long-term outcome. An open question remains about the link between possible biomarkers or polymorphisms, the long-lasting response, and the occurrence of severe adverse events (24).

There are no data about rechallenges of T-DXd after onset of severe and persistent thrombocytopenia or ILD ≥ grade 2 with consequent drug discontinuation. Identification of predictive factors of benefit and biomarkers of risk of toxicities should be investigated to define preventive strategy and personalized treatment in the precision medicine era (24).

Finally, neither of our two patients presented liver metastases which are known to be negative prognostic factors. Furthermore, liver metastases were associated with adverse tumor immune cell profiles and induction of immune response is one of the main mechanisms of action to HER2-targeted antibodies which affect significantly through immune activation (25).

Therefore, we questioned whether if these two patients had had liver metastases, the outcome would have been the same.

The next open question stands in the prognosis of our patients in case of disease relapse, but up to today, patients are free of disease and no oncologist would have thought at the start of T-DXd, nor at the time of toxicity onset.

## Data Availability

The original contributions presented in the study are included in the article/supplementary material, further inquiries can be directed to the corresponding author/s.
